# Distribution and abundance of human-specific *Bacteroides* and relation to traditional indicators in an urban tropical catchment

**DOI:** 10.1111/jam.12455

**Published:** 2014-02-25

**Authors:** J P Nshimyimana, E Ekklesia, P Shanahan, L H C Chua, J R Thompson

**Affiliations:** 1 Civil and Environmental Engineering, Massachusetts Institute of TechnologyCambridge, MA, USA; 2 Civil and Environmental Engineering, Nanyang Technological UniversitySingapore, Singapore

**Keywords:** *Bacteroides*, environmental contamination, source tracking, water quality

## Abstract

**Aims:**

The study goals were to determine the relationship between faecal indicator bacteria (FIB), the HF183 marker and land use, and the phylogenetic diversity of HF183 marker sequences in a tropical urban watershed.

**Methods and Results:**

Total coliforms, *Escherichia coli,* and HF183 were quantified in 81 samples categorized as undeveloped, residential and horticultural from the Kranji Reservoir and Catchment in Singapore. Quantitative-PCR for HF183 followed by analysis of variance indicated that horticultural areas had significantly higher geometric means for marker levels (4·3 × 10^4^ HF183-GE 100 ml^−1^) than nonhorticultural areas (3·07 × 10^3^ HF183-GE 100 ml^−1^). *E. coli* and HF183 were moderately correlated in horticultural areas (*R* = 0·59, *P* = 0·0077), but not elsewhere in the catchment. Initial upstream surveys of candidate sources revealed elevated HF183 in a wastewater treatment effluent but not in aquaculture ponds. The HF183 marker was cloned, sequenced and determined by phylogenetic analysis to match the original marker description.

**Conclusion:**

We show that quantification of the HF183 marker is a useful tool for mapping the spatial distribution and potential sources of human sewage contamination in tropical environments such as Singapore.

**Significance and Impact:**

A major challenge for assessment of water quality in tropical environments is the natural occurrence and nonconservative behaviour of FIB. The HF183 marker has been employed in temperate environments as an alternative indicator for human sewage contamination. Our study supports the use of the HF183 marker as an indicator for human sewage in Singapore and motivates further work to determine HF183 marker levels that correspond to public health risk in tropical environments.

## Introduction

Singapore is a highly urbanized island nation with limited freshwater resources. To protect urban catchments as water sources, the Public Utilities Board (PUB) of Singapore has embarked on the Active, Beautiful, Clean (ABC) Waters programme (PUB 2011). In this programme, green space and recreational infrastructure are brought together with stormwater management (PUB 2011) to both enhance public enjoyment and increase water security. In Singapore, reservoirs are the end-members of urban watersheds characterized by mixed land uses and extensive concrete-lined drainage systems. Water quality in these reservoirs is generally affected by nonpoint source pollution. Pollution of urban waters from human waste is one of the top concerns from a risk management perspective as numerous studies have established strong links between exposure to human waste and the spread of infectious disease (Pruss 1998). Faecal pollution is a major contributor to water quality degradation of urban beaches and water bodies worldwide (Pruss 1998; Johnson *et al*. 2003; Horman *et al*. 2004; Noble *et al*. 2006; USEPA 2009; Converse *et al*. 2011). Recent studies identify failing sewage infrastructure and a combination of sewage discharge, abundance of nutrients and favourable growth conditions as major factors influencing the persistence of faecal contamination in urban drainage systems (Field and O'Shea 1992; Rajal *et al*. 2007; Surbeck *et al*. 2010; Sauer *et al*. 2011). Tools to monitor and identify sources of human faecal contamination are critical for identifying and prioritizing management targets to improve urban water quality.

Faecal indicator bacteria (FIB), e.g. *Enterococci* and *Escherichia coli,* are widely used as proxies for the estimation of faecal contamination, yet their accuracy is limited by the inability to differentiate human and wildlife sources (Layton *et al*. 2010), variable correlation with human pathogens (Noble and Fuhrman 2001; Boehm *et al*. 2003, 2009; Horman *et al*. 2004), growth or persistence in the environment (Hardina and Fujioka 1991; Anderson *et al*. 2005; Yamahara *et al*. 2009, 2012) and errors associated with culture-based quantification such as cells that are dormant or viable but not culturable (VBNC) (Rahman *et al*. 1996; Menon *et al*. 2003). A major factor affecting the use of FIB in tropical areas is the ability of some strains to grow in warm, high nutrient environments (Hazen 1988; Rivera *et al*. 1988).

Nucleic acid-based techniques for detecting and enumerating FIB such as PCR and QPCR have been developed for emerging alternative indicators, circumventing biases associated with cultivation and allowing quantification of markers for which cultivation-based assays are not feasible (Bernhard and Field 2000a; Seurinck *et al*. 2005; Shanks *et al*. 2006; Bae and Wuertz 2009). The HF183 assay targeting the 16S rRNA gene of a human-associated *Bacteroides* strain has emerged as one of the most robust assays for identifying human sewage as this assay is highly specific for human faecal contamination (Bernhard and Field 2000a,b; Seurinck *et al*. 2005; Van De Werfhorst *et al*. 2011), with relatively few documented exceptions (McLain *et al*. 2009; Van De Werfhorst *et al*. 2011). The HF183 marker, originally described from a cultivation-independent study of microbial diversity in human faeces (Bernhard and Field 2000a), has since been detected in a cultivated bacterium, *Bacteroides dorei*. *B. dorei* is a strict anaerobe and is thus not expected to grow in oxic environments (Bakir *et al*. 2006). Thus, the HF183 marker, targeting *B. dorei*-like organisms, should qualify as a neutral tracer of human faecal contamination in surface waters (Bernhard and Field 2000a; Fogarty and Voytek 2005; Walters and Field 2009; Walters *et al*. 2009; Dick *et al*. 2010), and its use has been validated in various studies in temperate environments including in the United States (Bernhard and Field 2000b; Fogarty and Voytek 2005; Shanks *et al*. 2006; Santoro and Boehm 2007; Van De Werfhorst *et al*. 2011), Europe (Gawler *et al*. 2007), and Australia (Ahmed *et al*. 2008). Recent studies in Kenya (Jenkins *et al*. 2009), Tanzania (Pickering *et al*. 2010) and Bangladesh (Ahmed *et al*. 2010) have extended the use of the HF183 assay to tropical or semi-tropical conditions; however, the specificity of the marker for *B. dorei*-like organisms has not been confirmed by phylogenetic analysis nor has the marker abundance been compared to that of traditional indicators under tropical conditions. This study aims to address this research gap.

In this study, we have used detection and quantification of the HF183 marker for human-specific *Bacteroides* in parallel with *E. coli* and total coliforms to evaluate the potential distribution of sewage contamination in the watershed of Kranji Reservoir in Singapore. Previous work in the Kranji Reservoir Catchment has revealed high levels of FIB including *E. coli* and total coliforms, especially in the horticultural areas (NTU 2008); however, the distribution of human-specific *Bacteroides* is unknown. We have used cloning, sequencing and phylogenetic analysis to evaluate whether the HF183 marker recovered in Singapore matches the original marker description, and we provide a quantitative analysis of the correlation of HF183 abundance to that of *E. coli*, total coliforms and land-use characteristics in the reservoir. Our data support the use of HF183 marker quantification to improve the accuracy of human source identification in tropical environments; however, further work is needed to establish levels of the marker that correspond to sewage-associated risks in tropical environments such as Singapore.

## Materials and methods

### Site description

**Figure 1:**
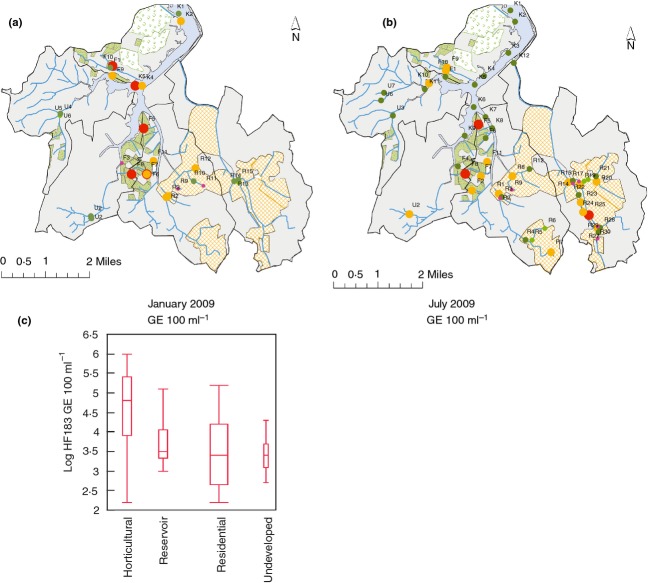
HF183 marker distribution and abundance in Kranji Reservoir and catchment. (a) and (b) Abundance of the HF183 marker in Kranji Reservoir and catchment as determined by quantitative PCR. (c) HF183 marker abundance in each land-use category showing median, upper and lower quartiles spanning maximum and minimum observations. Bar width is proportional to the number of samples in each category. (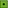) Presence of total Bacteroides; (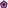) Inhibited Samples and (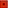) Presence of HF183. HF183—GE 100 ml^−1^: (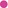) <150; (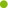) 151–1000; (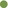) 1001–10 000; (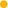) 10 001–100 000; (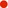) 100 001–1 000 000. (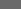) and Farming/Horticultural; (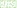) Recreational; (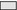) Undeveloped; (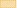) Residential; (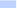) Kranji Reservoir.

The Kranji Reservoir catchment (Fig.1) located in the northwest of Singapore island (1°25′N, 103°43′E) drains 61 square kilometres (NTU 2008). The catchment is one of the most diversified watersheds in Singapore in terms of land use, containing areas designated as ‘residential’—dominated by high-density high-rise buildings, ‘undeveloped’—characterized by native vegetation and low population density and ‘farming/horticultural’—dominated by cultivation of flowers, vegetables, fish and chickens. The watershed is served by a system of concrete-lined drains that convey stormwater runoff from the watershed to the Kranji Reservoir. The residential sites are sewered, while horticultural sites are served by on-site treatment plants. The Kranji Reservoir is an impoundment reservoir with an estimated capacity of 16 million cubic metres based on area and mean depth. Drinking water is purified through Singapore's advanced water treatment systems before distribution (NTU 2008). The climate in Singapore is tropical with temperatures ranging from diurnal highs of 29–31°C to lows of 23–24°C.

### Sample collection and DNA extraction

Water samples were collected in the Kranji Reservoir and surrounding catchment (Fig.1) during the months of January and July 2009. Water samples were collected from concrete-lined drainages near catchment monitoring stations of residential (R), farming/horticultural (F) and undeveloped (U) areas, which represent 19, 5 and 76% of catchment land use, respectively (Chassard *et al*. 2007). Samples were also collected from Kranji Reservoir (K) during January and July 2009. Samples were collected during dry weather, with the exception of seven samples from July 2009 that were collected from high water flows following a rain event (R1, R8, R12, F9, F10, F11 and U3). During January 2010, samples were collected from sites within the horticultural area suggested as potential sources of faecal contamination by local experts. Most of the horticultural areas are un-sewered and are served by on-site wastewater treatment plants and thus are potential sources of faecal contamination. Samples were collected from fish ponds (near F4 and F7) and effluents from several on-site wastewater treatment facilities (near F5, F7, F8 and F9/F10). Finally, a raw sewage sample from a sewer system in a high-density residential area was collected for comparison.

Water samples were collected in sterile 500 ml Whirl-Pak® bags (Nasco, Fort Atkinson, WI, USA) or 1L Nalgene bottles and immediately stored on ice. Reservoir water samples were collected at a depth of 1 m using an AquaStore Model 1010 Niskin water sampler (AquaStore, Aquatic Network, Miami, FL, USA). Microbiological analysis (total coliforms and *E. coli*) and filtration of water samples onto a Millipore Sterivex™-GS 0·22 *μ*m Filter Unit (Millipore, Billerica, MA, USA) were accomplished within 6 h of sample collection. Filters containing biomass were stored at −80°C until further analysis.

For preparation of DNA from environmental samples, membranes were aseptically removed from Sterivex filter cartridges, split in half and sliced into 8–10 strips using a flame-sterilized blade and forceps. DNA for PCR and cloning were extracted from one half-filter with the Ultraclean™ Soil Isolation Kit (MO BIO Laboratories, Carlsbad, CA, USA), and the remaining half-filter was stored frozen for future analyses. DNA for QPCR was subsequently extracted from the second half of the filter using the Ultraclean Plant DNA Kit (MO BIO Laboratories) that adds an additional reagent for removal of plant-based PCR inhibitors, such as may be associated with high algal biomass found at multiple sites in the catchment. Kits were used according to the manufacturer's protocols that include a bead-beating step to remove biomass from the membrane and to mechanically lyse cells. DNA samples, eluted in 50 *μ*l buffer, were electrophoresed on 1% agarose and quantified on a NanoDrop® ND-1000 Spectrophotometer (NanoDrop Technologies, Wilmington, DE, USA) to validate their quality and purity. Environmental DNA samples were kept on ice during extraction procedures, and DNA samples were stored at −20°C.

### Detection of total and human *Bacteroides* by PCR

Initial amplification of the HF183 marker by conventional PCR (Bernhard and Field 2000a,b) revealed a high percentage of PCR-inhibited samples and dilution of samples to relieve PCR inhibition resulted in a loss of sample signal and undetectable PCR yields. Successful and reproducible amplification was achieved with two modified approaches. First, cycling conditions adapted from Bernhard and Field (Bernhard and Field 2000a,b) were modified with an initial 10-cycle ‘touchdown’ annealing step (Fogarty and Voytek 2005)**.** Amplification with HF183 marker primers HF183F and 708R (Table1) was carried out in a volume of 50 *μ*l with 15–275 ng DNA, 25 *μ*M of each primer, 10 mM of deoxynucleoside triphosphate, 1·25 units of *Taq* DNA Polymerase, 10X ThermoPol Reaction Buffer and autoclaved Mill-Q-water. The PCR cycling conditions were as follows: 3 min at 95°C; followed by 10 cycles of 30 s at 95°C, 30 s at 63°C decreasing 1°C each cycle and 30 s at 72°C. This was followed by 40 cycles of 30 s at 95°C, 30 s at 53°C, then 90 s at 72°C; concluding with a final extension of 7 min at 72°C. Secondly, a semi-nested PCR protocol was used to detect both the *Bacteroides-Prevotella* group and the human-specific *Bacteroides* HF183 marker at a higher sensitivity (Shanks *et al*. 2006). PCR mixtures for the first and second stages of the semi-nested protocol contained 25 *μ*M of each primer, 10 *μ*M of dNTPs, 0·625 units of *Taq* DNA Polymerase, 10X ThermoPol Reaction Buffer and autoclaved Mill-Q-water. For the first stage, primers Bac32F and Bac708R (Table1) were used to amplify *Bacteroides-Prevotella* using cycling conditions of 3 min at 95°C; followed by 35 cycles of 30 s at 95°C, 30 s at 53°C and 1 min at 72°C; followed by a final 3 min at 72°C. Positive amplicons were then purified (QIAquick® PCR Purification Kit, QIAGEN®, Valencia, CA, USA) and used as template for a second round of amplification with primers HF183F and Bac708R using the same cycling conditions with an annealing temperature of 63°C. Each PCR included a positive control plasmid (pHF183) containing an HF183 marker sequence (16S rRNA positions 183–708) from an uncultured *Bacteroides* cloned into a PCR2.1 TOPO plasmid (provided by A. Boehm). No template negative controls were propagated through all PCR steps and confirmed to be clean.

**Table 1:** Primers used

Primer	Target	Sequences (5′–3′)	References
Bac32F	*Bacteroides-Prevotella* group	AACGCTAGCTACAGGCTT	Bernhard and Field (2000a)
Bac708R	*Bacteroides-Prevotella* group	CAATCGGAGTTCTTCGTG	Bernhard and Field (2000a)
HF183F	HF183 marker	ATCATGAGTTCACATGTCCG	Bernhard and Field (2000a)
Bac242R	Bacteroidales 16S rRNA	TACCCCGCCTACTATCTAATG	Seurinck *et al*. (2005)

### Clone library preparation, sequencing and phylogenetic analysis

PCR products from Kranji Reservoir (K6) (semi-nested protocol) and from farming/horticultural (F5) and residential areas (R15) (touchdown protocol) were gel-purified (QIAquick®; QIAGEN, Valencia, CA, USA) and cloned using the Zero Blunt® TOPO® kit (Invitrogen™, Grand Island, NY, USA). Sequencing was performed uni-directionally on an ABI3700 using HF183F as a sequencing primer. Sequences were assembled into operational taxonomic units (OTUs) at >99% identity using Sequencher 4.010.1 (Gene Codes, Ann Arbor, MI, USA). Closely related sequences from other studies were identified using NCBI-BLAST and were aligned to OTUs using ClustalX (Thompson *et al*. 1997). Phylogenetic relationships between sequences were reconstructed ClustalX implementing the neighbour-joining method. Nucleotide sequences have been deposited at NCBI with accession numbers KC492830–KC492832.

### QPCR and quantification of DNA extraction efficiency and PCR inhibition

#### Quantitative polymerase chain reaction

The human-specific HF183 marker was quantified by QPCR using primers HF183F and 242R (Table1) (Seurinck *et al*. 2005) using the LightCycler® 480 Real-Time PCR system and software v. 1.5.0 (Roche Applied Sciences, Indianapolis, IN, USA) for calculation of crossing point (Cp) values and melting temperature (Tm) analysis. The reverse primer for QPCR described by (Seurinck *et al*. 2005) allowed formation of a 83-bp amplicon compatible with QPCR and was confirmed to match HF183 marker sequences recovered using the HF183F 708R primer pair from clone libraries in this and other studies. QPCR reaction mixtures consisted of 10 *μ*l of KAPA SYBR® FAST 2X Master Mix (KAPABIOSYSTEMS, Woburn, MA, USA), 10 *μ*M of each primer and 1 *μ*l DNA template. Amplification followed the manufacturer's instructions; briefly, reactions were subjected to a pre-incubation step of 95°C for 3 min, followed by 50 cycles of 95°C for 10 s, 53°C for 20 s and 72°C for 1 s. Each sample was analysed in triplicate, and Cp values were examined after amplification to verify consistency (i.e. coefficient of variation ≤3%). To confirm the specificity of amplification, melting temperatures (Tm) of sample amplicons were confirmed to be within two standard deviations of the mean Tm associated with QPCR standards at concentrations of 10^1^–10^6^ copies per QPCR (78·93°C ± SD 0·15), while late-stage PCR artefacts were associated with standards at concentrations exceeding >10^7^ copies QPCR^−1^ (Tm 80·01°C ± SD 1·92). Tenfold serial dilutions of plasmid DNA containing the HF183 marker were used to generate a standard curve of Cp values versus target DNA concentration for each QPCR run using a least-squares fit. Confidence intervals of predicted target concentrations based on measured Cp values were calculated based on propagation of error in the standard curve (Harris 1995). The limit of detection (LOD) for each 96-well plate was determined based on uncertainty in the standard curve as the upper 99th per cent confidence interval of the Cp values of the negative controls or 50 cycles if no signal was apparent. For consistency in statistical analysis, the highest LOD used to indicate nondetect of the HF183 marker was selected as the study-wide LOD. The amplification efficiency (E) for each QPCR run was calculated from the slope of the standard curve and was consistently in the range of 99–100%.

We determined the impact of inhibition on QPCR by spiking 1·2 × 10^5^ copies of the positive control plasmid (pHF183) bearing the HF183 marker into an aliquot from each sample before QPCR amplification and comparing the results measured by QPCR with and without spike addition. If the spiked sample was quantified as having less than 65% of the added amount of HF183 marker (corresponding to both the 95% confidence interval for quantification of the QPCR standard curve and observed variability between technical replicates), then the sample was diluted tenfold and re-analysed.

#### Estimation of HF183 marker genome equivalents (GE) in natural waters

To convert from QPCR-detected HF183 marker copies to units of genome equivalents (GE), the efficiency of DNA extraction from water-borne biomass concentrated onto filters was determined. *Bacteroides dorei* strain DSM 17855, which contains a single-copy 16S rRNA gene sequence (Bakir *et al*. 2006) matching the HF183 marker, was obtained from the German Collection of Microorganisms and Cells (DSMZ, Braunschweig, Germany) and grown to stationary phase in a modified PYG-Medium at 37°C under anaerobic conditions for 4 days. Cells were pelleted from 1 ml aliquots of *B. dorei* culture (7000 g for 10 min) and washed in phosphate-buffered saline (PBS) to reduce cell-free DNA. Replicate cell pellets were either resuspended in DNA extraction buffer and subjected to the extraction protocol directly or were resuspended with or without 100-fold dilution into natural water samples (200 ml) obtained from the Charles River, MA, which is adjacent to the laboratory where the sample analysis was performed. River water samples with and without spiked cells were then concentrated by filtration onto Millipore Sterivex™-GS 0·22 *μ*m Filter Units (Millipore, Billerica, MA, USA). Nucleic acids were extracted from cell pellets and filters with or without spiked cells using the UltraClean Plant DNA Isolation Kit (MO BIO Laboratories Inc., Carlsbad, CA, USA). Extracted samples were subjected to analysis by QPCR to determine the extraction efficiency (E) calculated as the ratio of HF183 marker recovered from filters relative to the values obtained from quantification of HF183 marker copies in *B. dorei* cell pellets subjected to direct extraction. After determination of DNA extraction efficiencies, genome equivalents (GE) of the *B. dorei* HF183 marker in environmental samples were determined as: 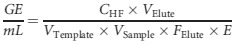
where *C*_HF_ is the number of HF183 marker copies detected by the QPCR (copies QPCR^−1^); *V*_Elute_ is the volume of buffer in which DNA extracts are suspended following purification (*μ*l); *V*_Template_ is the volume of DNA extract added to the QPCR (*μ*l); *V*_Sample_ is the volume of environmental sample subjected to DNA extraction (*μ*l); *F*_Elute_ is the fraction of sample DNA that is eluted, accounting for known volumetric losses in the extraction protocol (=0·75); and E is the efficiency of HF183 marker recovery from *B. dorei* cells suspended in river water and accounts for the use of half-filters in DNA extraction. Error associated with estimation of genome equivalents (GE) was determined by propagation of random error through multiplicative expressions based on standard methods (Harris 1995). Error tolerance for volumetric measurement was ±1%, while relative errors for *C*_HF_ and E were determined using measured standard deviations.

### Enumeration of total coliforms and *E. coli*

Total coliforms (TC) and *E. coli* bacteria in Kranji Reservoir and catchment were enumerated in January and July 2009, and January 2010 using the Hach m-ColiBlue24® method (Hach Company, Loveland, CO, USA) and Colilert Quanti-Tray®/2000 **(**IDEXX Laboratories, Westbrook, ME, USA), respectively. Sample dilution was performed to increase the range of *E. coli* and TC abundances quantified.

### Statistical analysis of the distribution of human *Bacteroides*, *E. coli,* and TC

Sampling sites, land-use categories and abundance of bacterial markers were mapped with ArcGIS version 10.1 software (ESRI®, Redlands, CA, USA). Abundance data for *E. coli* and TC (CFU 100 ml^−1^ or MPN 100 ml^−1^) and the HF183 marker (GE 100 ml^−1^) were log_10_-transformed to achieve normal distributions and meet the assumptions of a parametric test (Srinivasan *et al*. 2011). Two-way analysis of variance (ANOVA) followed by *post hoc* Tukey's HSD tests was calculated in JMP Pro v.10 (SAS Institute Inc., Cary, NC, USA) to determine whether indicator abundance varied across sampling dates and land-use categories. The relationship between log_10_-transformed indicator bacteria (*E. coli* and total coliforms) and HF183 marker levels across land-use categories was examined by Pearson's correlation and hierarchical clustering using Ward's method on standardized data (JMP Pro v.10). Samples harbouring the HF183 marker (HF), *E. coli* (EC) or total coliforms (TC) at or below the detection limit (i.e. <150 HF GE 100 ml^−1^; <1 CFU or MPN 100 ml^−1^ for EC and TC) were represented in correlation and clustering analyses as the detection limit. To avoid biases associated with sampling method, samples with indicator levels above the detection maxima (i.e. TC > 4 × 10^7^ CFU 100 ml^−1^ or EC > 4 × 10^6^ CFU 100 ml^−1^ in January 2009 and TC > 1·3 × 10^6^ MPN 100 ml^−1^ or EC > 1·55 × 10^5^ MPN 100 ml^−1^ in July 2009) were represented by the lower MPN-based detection maximum.

## Results

### Distribution and phylogenetic analysis of human *Bacteroides* in the Kranji Reservoir and catchment

The HF183 assay was used to detect the human *Bacteroides* marker throughout the Kranji Reservoir and catchment. Due to the need to dilute samples to reduce PCR inhibition, Touchdown PCR (detection limit 1000 copies per PCR) was unreliable for quantitative assessment of presence/absence. When the semi-nested PCR protocol was applied (detection limit 10 copies per PCR), a high proportion of samples was found to be positive for the *Bacteroides-Prevotella* group (96% in January 2009 and 100% in July 2009) and the HF183 marker (83% in January 2009 [*n* = 20/24] and 100% in July 2009 [*n* = 30/30]) (Table2).

**Table 2:** Extraction efficiency for recovery of DNA off Sterivex filters for *Bacteroides dorei* spiked into freshwater

Samples (Sample Volume)	Number of Samples	HF183 copies per QPCR ± SD	Recovery Efficiency ± SD (%)
River water (200 ml)	6	8·3 × 10^4^ ± 2·5 × 10^4^	–
*B. dorei* culture* (1 ml)	3	8·6 × 10^9^ ± 9·5 × 10^8^	–
River water (200 ml) spiked with *B. dorei* culture* (1 ml)	6	4·8 × 10^9^ ± 1·8 × 10^9^	56 ± 21
River water (200 ml) spiked with 1:100 dilution of *B. dorei* culture* (1 ml)	6	5·2 × 10^7^ ± 2·5 × 10^7^	61 ± 29
All spiked samples	12	–	58 ± 24

* The *Bacteroides dorei* culture optical density (OD 600 nm) was 1·85.

**Figure 2:**
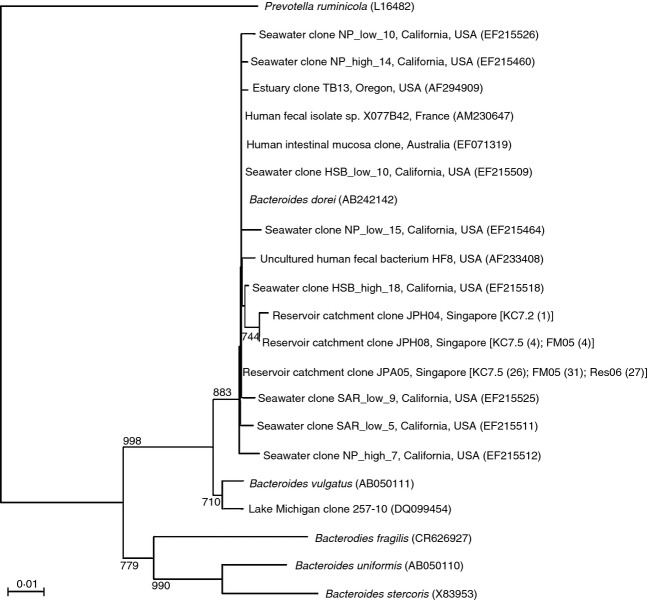
Neighbour-joining tree of cloned sequences recovered from Kranji Reservoir and catchment samples using the human *Bacteroides*-specific HF183F and 708R primer pair. For each representative sequence, the number of sequences sharing >99% nucleotide identity from each of the three field sites is provided in brackets. Field sites are: R15–Residential Area, Bukit Panjang; F5–Farm Area in Tengah; K6–Kranji Reservoir at branch point between catchment and open water. The most closely related reference sequences included in the phylogenetic analysis were downloaded from the NCBI database (9/16/10—12/15/2009) and correspond to the following studies: EF215526, EF215460, EF215509, EF215464, EF215518, EF215525, EF215511, EF215512 from (Santoro and Boehm 2007); AF233408 and AF294909 from (Bernhard and Field 2000a,b).

Clone libraries of HF183 marker sequences (16S rRNA gene positions 183–708) were prepared from three samples obtained from sites with different land-use designations: Farming/horticultural (F5—1/2009), residential (R15—1/2009) and reservoir (K6—7/2009). Sequencing 36 clones per library yielded a total of 93 good quality sequences. Clustering analysis revealed three closely related operational taxonomic units (OTUs) defined as a set of sequences with >99% nonambiguous nucleotide sequence identity. One sequence type (JPA05) was observed in the majority at all three sites and corresponded to the nucleotide sequence of the *B. dorei*-type strain. A second sequence type (JPH08) was observed at two sites (R9, *n* = 4; F5, *n* = 4). The third sequence type (JPH04) was detected once (R15, *n* = 1). Phylogenetic analysis of the HF183 marker sequences (Fig.2) revealed that HF183 marker sequences obtained from this study were closely related to those from various other studies (Paster *et al*. 1994; Ruimy *et al*. 1996; Bernhard and Field 2000b; Miyamoto *et al*. 2000; Bower *et al*. 2005; Cerdeno-Tarraga *et al*. 2005; Bakir *et al*. 2006; Chassard *et al*. 2007; Santoro and Boehm 2007).

### Validation of QPCR assay for determination of HF183 marker genome equivalents

Genome equivalents of the HF183 marker were determined in samples from the Kranji Reservoir and catchment by QPCR. Quantification of the HF183 marker by QPCR was linear over the range of 10^1^–10^8^ HF183 marker copies per QPCR (*R*^2^ > 0·99). HF183 marker copies detected by QPCR were converted to genome equivalents (GE) based on a measured DNA extraction efficiency of 58% ± 24% for *B. dorei* suspended in natural freshwater (Table2). Detection limits for the HF183 marker were determined for each 96-well QPCR run based on error from the respective run's standard curve. Single-run detection limits varied from <10 to 19 copies per QPCR (*P* < 0·01) which, after adjustment for sample volumes, corresponded to a conservative detection limit of 150 GE 100 ml^−1^ which was set as the study-wide LOD for subsequent statistical analyses.

### HF183 marker in the Kranji Reservoir and catchment

Quantification of HF183 marker genome equivalents in samples from the Kranji Reservoir and catchment revealed a wide range of abundance from <150 GE 100 ml^−1^ to 9·7 × 10^5^ GE 100 ml^−1^ (Fig.1a,b, and S1). Sites within the designated horticultural areas were associated with the highest levels of HF183 marker with a geometric mean of 6·0 × 10^4^ GE 100 ml^−1^ and 3·2 × 10^4^ GE 100 ml^−1^ for January and July 2009, respectively (Fig.1c, S1). Significantly lower levels of the HF183 marker were observed in the nonhorticultural areas (geometric mean 3·1 × 10^3^ GE 100 ml^−1^) where geometric means for residential, undeveloped and reservoir were 2·5 × 10^3^ GE 100 ml^−1^, 2·7 × 10^3^ GE 100 ml^−1^, and 5·2 × 10^3^ GE 100 ml^−1^, respectively (Fig.1c). Two-way analysis of variance (anova) revealed differences in mean log-transformed HF183 levels with land-use category (*F* = 8·80; *P* < 0·0001) but not sample date or the interaction of date and land use (*F* = 0·699; *P* = 0·41 and *F* = 1·32; *P* = 0·27, respectively). Farming/horticultural areas had significantly elevated HF183 levels relative to residential and undeveloped areas and the reservoir (Tukey's HSD α < 0·05) (Table3).

**Table 3:** Distribution of *Bacteroides-Prevotella* and HF183 in Kranji catchment, Singapore

Land Use	Date	Total Sites	Incidence Bacteroides-Prevotella*	Incidence HF183*	Log10 HF183 GE 100 ml^−1^ (SD)	anova†		
						HF183	*Escherichia coli*	TC
Farming/horticultural (F)	Jan. 09	9	89% (9)	78% (9)	4·78±0·04	A	A	A
		July 09	10	100% (10)	100% (9)				4·50±0·05
		Total	19	95% (19)	89% (19)				4·63±0·05
Residential (R)	Jan. 09	9	100% (8)	88% (8)	3·42±0·09	B	B	A, B
		July 09	28	100% (7)	100% (7)				3·41±0·16
		Total	37	100% (15)	93% (15)				3·40±0·12
Undeveloped (U)	Jan. 09	5	100% (2)	100% (2)	3·40±0·15	B	C	B, C
		July 09	4	100% (3)	100% (3)				3·73±0·18
		Total	9	100% (5)	100% (5)				3·44±0·16
Kranji Reservoir (K)	Jan. 09	4	100% (4)	75% (4)	4·40±0·07	B	C	C
		July 09	12	100% (11)	100% (11)				3·57±0·12
		Total	16	100% (15)	93% (15)				3·78±0·09

* Determined by semi-nested PCR. Numbers in parentheses correspond to samples included in the presence/absence analysis.

† Land-use groups differentiated by the *post hoc* Tukey's honestly significant difference (HSD) test (α < 0·05) are designated by distinct letters.

TC, total coliform.

### Correlation between human *Bacteroides,* total coliforms, and *E. coli*

To examine the hypothesis that *E. coli* and the HF183 marker were both predictive for the presence of human sewage, we tested the relationship between *E. coli*, total coliforms and land use/sample date and compared log-transformed concentrations of HF183, *E. coli,* and total coliforms across the dataset with the expectation that positive correlations would be consistent with prediction of the same property. Similar to the ANOVA results obtained for the HF183 marker, two-way analysis of variance revealed differences in mean log-transformed *E. coli* levels with land-use category (*F* = 26·3; *P* < 0·0001) but not sample date or the interaction of land use and sample date (*F* = 2·86; *P* = 0·09 and *F* = 0·701; *P* = 0·55, respectively), while total coliform levels varied with land-use category (*F* = 15·73; *P* < 0·0001), sample date (*F* = 5·48; *P* = 0·0220) and the interaction of land use and sample date (*F* = 3·65; *P* = 0·0164) (Table3). Farming/horticultural areas had significantly elevated *E. coli* and total coliform levels relative to undeveloped areas and the reservoir (Tukey's HSD alpha <0·05). In contrast to the HF183 marker and *E. coli,* total coliform levels in the farming/horticultural areas were not significantly different from levels in the residential areas.

**Figure 3:**
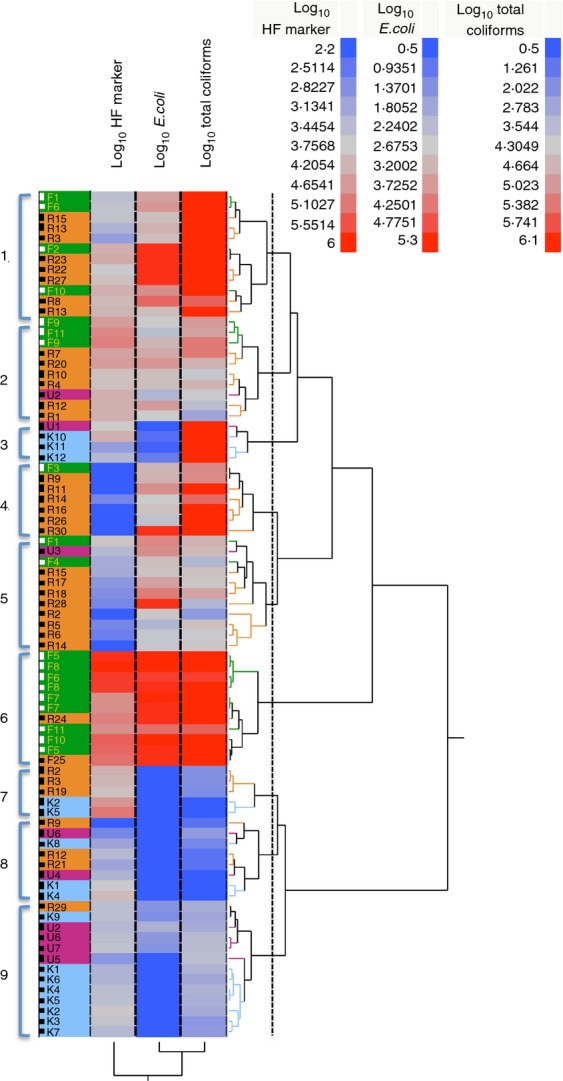
Hierarchical clustering of log_10_-transformed indicator concentrations (HF–HF183 marker, EC*—Escherichia coli*, and TC–total coliforms). Colours in left-hand column denote land-use categories. Branch lengths correspond to distances calculated by Ward's method. Dendrogram calculated in JMP Pro v.10. (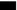) January 2009; (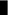) July 2009; (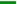) Horticultural; (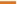) Residential; (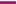) Undeveloped and (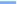) Reservoir.

We observed a weak but significantly positive correlation in the total dataset between HF183 marker levels and *E. coli* (*R* = 0·34; *P* = 0·0014) that was driven by moderate correlation of these indicators within the farming/horticultural areas (*R* = 0·59; *P* = 0·0077). The majority of samples collected from the farming/horticultural areas had elevated levels of all indicators and emerged as a distinct cluster in hierarchical clustering analysis of indicator profiles (Fig.3, cluster 6). In the nonhorticultural areas (residential, undeveloped and within the reservoir), correlation between HF183 and *E. coli* or total coliforms was weak and not significant (*P* > 0·05) (Table4). Some samples with *E. coli* levels exceeding the USEPA single-sample limit had below study median or nondetected HF183 levels (Fig.3, clusters 4 and 5), while in several samples near-median to above-median HF183 levels were found in samples that had compliant *E. coli* levels (Fig.3, clusters 3 and 7). The majority of samples collected from the reservoir were characterized by low levels or nondetection of all indicators (Fig.3, cluster 9). HF183 marker levels at 21 sites sampled during both January 2009 and July 2009 showed consistency across sampling dates (*R* = 0·62; *P* = 0·0025).

**Table 4:** Correlation of log10-tranformed HF183 marker abundance to total coliform (TC) or *Escherichia coli* concentrations

Correlations	Pearson Coefficients (*n*)		
	TC and HF	*E. coli* and HF	HF_Jan_ and HF_July_
Jan-09	0·044 (27; *P* = 0·83)	0·34 (27; *P* = 0·085)	–
Jul-09	**0·36** (54; *P* = 0·0075)	**0·39** (54; *P* = 0·0038)	–
Jan & July 09	0·19 (81; *P* = 0·080)	**0·34** (81; *P* = 0·0014)	**0·62** (21; *P* = 0·0025)
Farming/horticultural	**0·47** (19; *P* = 0·042)	**0·59** (19; *P* = 0·0077)	0·38 (8; *P* = 0·35)
Residential	0·043 (37; *P* = 0·80)	0·17 (37; *P* = 0·30)	0·29 (7; *P* = 0·51)
Undeveloped	0·47 (9; *P* = 0·2030)	0·34 (9; *P* = 0·37)	NA (2)
Reservoir	−0·43 (16; *P* = 0·098)	−0·11 (16; *P* = 0·69)	0·57 (4; *P* = 0·43)

Bold highlights denote significant correlations (*P* < 0·05).

### Investigation of potential sources

**Figure 4:**
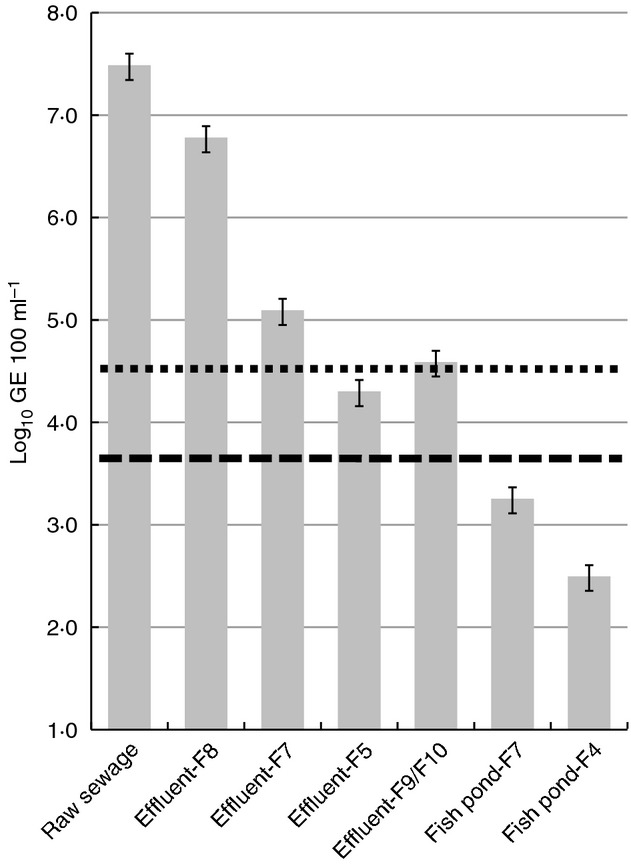
Quantification of HF183 marker levels in candidate sources of human faecal contamination within the farming/horticultural areas of Kranji catchment. Horizontal lines denote geometric means of samples from horticultural (dotted) and reservoir areas (dashed).

To gain preliminary insight into potential sources of human faecal contamination in the horticultural areas, we analysed effluent samples from four on-site wastewater treatment systems (near sites F8, F7 and F5, and between F9 and F10), from two fish ponds (near sites F7 and F4) and a sample of raw sewage from the sanitary sewer of a residential area. The raw sewage sample contained the HF183 marker at an abundance of 3·1 × 10^7^ GE 100 ml^−1^ which is similar to the range of levels observed associated with sewage in other studies (i.e. 4·0 × 10^6^ to 2·5 × 10^8^ HF183 marker copies 100 ml^−1^) (Van De Werfhorst *et al*. 2011). Samples of effluent from the fish ponds and three of four on-site wastewater treatment systems revealed HF183 marker concentrations within the range of variability observed in the catchment (Fig.4). However, one effluent (Effluent–F8) connected to farming/horticultural site F8 had elevated HF183 marker levels similar to that observed in the raw sewage sample and was identified as a concentrated source of sewage contamination (Fig.4).

## Discussion

Maintenance of high water quality is necessary to enable recreational activities such as fishing and boating and to protect drinking water resources. However, identifying the primary mechanisms that introduce human sewage into reservoirs and drainage systems is a challenge, especially in tropical systems where the conservative behaviour of sewage indicators has not been well established. In this study, we compared the abundance and distribution of FIB (*E. coli* and total coliforms) with the distribution and abundance of an alternative DNA-based human faecal indicator, the HF183 marker, to evaluate the distribution of human sewage contamination in the Kranji Reservoir and catchment. Semi-nested PCR revealed that the human-specific HF183 marker was widespread in the Kranji Reservoir and its catchment (Table2), however, did not provide information regarding the relative abundance of the marker across different sites or potential existence of a low, but nonzero, environmental-baseline abundance. Analysis of sequences recovered with the HF183F 708R primer pair confirmed that the HF183 assay was specific for *Bacteroides dorei*–like organisms in Singapore and similar to sequences recovered from previous studies designed to validate the assay (Fig.2). Although we cannot rule out contribution of HF183 marker from alternate sources in this environment, previous studies have revealed low cross-reactivity of the HF183 marker, as amplified with the 183F-708R primer pair, with nonhuman sources (Bernhard and Field 2000a; McLain *et al*. 2009; Van De Werfhorst *et al*. 2011).

The use of the Sybr HF183 QPCR assay which relies on primer pair 183F-242R to generate a suitable sized amplicon for QPCR subsequently revealed differences in HF183 marker abundance among sites that were correlated with land use (Fig.1a–c) and pointed to on-site treatment plants within horticultural areas as potential contributors to elevated levels of human faecal contamination. Melting temperature profiles of QPCR amplicons in our study supports amplification of a single 16S rRNA sequence type, corresponding to sequenced clones and supporting the specificity of the QPCR assay for quantification of the same bacterial group as detected with the 183F-708R primer pair. *E*. *coli* and total coliforms were also elevated in the horticultural areas and were correlated with HF183 marker levels (Fig.3, cluster 6). In contrast, in the nonhorticultural areas, high *E*. *coli* concentrations were not well correlated with the HF183 marker, possibly due to nonconservative behaviour of *E. coli* as a tracer for human sewage (Fig.3, clusters 1, 4, 5). The single-sample threshold recommended by the USEPA for *E. coli* in freshwater used for water-contact recreation is 235 100 ml^−1^ (USEPA 1986, 2002a, 2012). A similar threshold has not been established for the HF183 marker (Ashbolt *et al*. 2010); however, for this specific environment, we sought to identify potential sources for human sewage contamination by identifying samples with HF183 levels above baseline levels observed in the reservoir and catchment. In the farming/horticultural areas, 18 of 19 samples had *E*. *coli* levels above the USEPA single-sample limit and of these 72% (13/18) also had HF183 levels higher than the study median (i.e. >4·16 × 10^3^ GE 100 ml^−1^). In contrast, in the nonhorticultural areas, 30 samples (of 62 total) exceeded the USEPA single-sample threshold for *E. coli*, but only 7/30 (23%) of these also had HF183 levels above the study median. This raises the possibility that many of the nonhorticultural sites with elevated *E. coli* levels may not be appreciably contaminated by human sewage.

Previous studies of waterways in temperate urban environments suggest an absence of correlation between HF183 marker and other indicator bacteria (Converse *et al*. 2011; Sauer *et al*. 2011). These earlier results are consistent with our observations of poor correlation between *E. coli* and HF183 marker in the nonhorticultural areas of the Kranji catchment. *E. coli*'s ability to serve as a FIB is hampered by the potential for environmental growth (Hazen 1988; Rivera *et al*. 1988; Hardina and Fujioka 1991), especially in warm tropical areas. Thus, the HF183 marker, which is not expected to grow under oxic conditions, may act as a more specific marker for human sewage in this catchment. However, it is not known if HF183 marker-bearing organisms can proliferate in environmental microhabitats that mimic the gut—i.e. are warm, anoxic and nutrient-rich. Such microhabitats may be present in the Kranji catchment and proliferation of HF183-marker-bearing organisms under eutrophic tropical conditions needs to be better constrained before the HF183 marker is adopted as a reliable standard in tropical areas (Balleste and Blanch 2010; Surbeck *et al*. 2010).

Agricultural areas in the United States and elsewhere are frequently associated with water quality impairments due to nutrient loading and agricultural wastes (USGS 1999). In Singapore, where farming activities are dominated by vegetable and flower horticulture, aquaculture and a very limited amount of chicken farming, combined drainage systems that merge sanitary wastewater with farm wastewater, and stormwater into the same discharge drain may contribute to impaired water quality (Chua *et al*. 2010). Indeed, our partial survey of potential sources of contamination in the farming/horticultural area pointed to a wastewater effluent as a source of human faecal contamination. In addition, direct discharge of septic tanks into the main drainage was observed at sites F8 and F7, and a make-shift toilet in use by farm workers and discharging into a surface-water drain was observed at site F6. These observations provide additional ground truthing of the utility of the HF183 marker assay to identify sources of human wastes in a complex environmental background.

The farming/horticultural areas in the Kranji catchment are located close to the reservoir and many farms drain almost directly into the reservoir. Although not necessarily producing high-volume flows, the drains exhibit very high coliform bacteria concentrations (Bossis 2011) and thus deliver a significant bacterial load to the reservoir. The effects on the reservoir were observed in water-quality samples collected on a north–south transect along the reservoir (Zhang 2011). These showed *E. coli* concentrations in excess of 100 MPN 100 ml^−1^ and as high as 5,000 MPN 100 ml^−1^ in the middle upstream arm of the reservoir adjacent to the horticultural areas as compared to single-digit *E. coli* concentrations in the main body of the reservoir. This is consistent with our results, in which reservoir sites K2 and K5 were associated with above-median levels of the HF183 marker in January 2009 (but not July 2009).

This study was carried out to determine the distribution of HF183 marker in a mixed tropical urban environment, to identify potential sources of human faecal contamination to the Kranji Reservoir and to phylogenetically validate the use of the HF183 marker in Singapore and similar tropical urban environments. Based on a synthesis of these results, we conclude that quantification of the HF183 marker targeting bacteria closely related to *B. dorei* can be a useful tool for mapping the spatial distribution of human sewage contamination and identifying potential sources of human sewage contamination in tropical environments such as Singapore. However, further studies are needed to understand the ecology of organisms bearing the HF183 marker in tropical environments, to confirm that they act as conservative tracers of human faecal contamination and to relate these levels to human health risks.

## Supporting Information

Figure S1 HF183 marker genome equivalents (GE 100 ml^−1^) in Kranji Reservoir and catchment samples collected in January 2009 (a) and July 2009 (b). *denotes samples with HF levels below detection (i.e. <150 GE 100 ml^−1^). Sample and colour codes: undeveloped area (U) (purple bars), farming/horticultural area (F) (green bars), residential area (R) (orange bars), Kranji Reservoir (K) (blue bars). Error bars correspond to standard deviations calculated through uncertainty propagation.

**Table S1** Quantification of Indicator Bacteria in Samples from Kranji Reservoir Catchment, Singapore.

## References

[b1] Ahmed W, Stewart J, Powell D, Gardner T (2008). Evaluation of Bacteroides markers for the detection of human faecal pollution. Lett Appl Microbiol.

[b2] Ahmed W, Yusuf R, Hasan I, Goonetilleke A, Gardner T (2010). Quantitative PCR assay of sewage-associated Bacteroides markers to assess sewage pollution in an urban lake in Dhaka, Bangladesh. Can J Microbiol.

[b3] Anderson KL, Whitlock JE, Harwood VJ (2005). Persistence and differential survival of fecal indicator bacteria in subtropical waters and sediments. Appl Environ Microbiol.

[b4] Ashbolt NJ, Schoen ME, Soller JA, Roser DJ (2010). Predicting pathogen risks to aid beach management: the real value of quantitative microbial risk assessment (QMRA). Water Res.

[b5] Bae S, Wuertz S (2009). Rapid decay of host-specific fecal Bacteroidales cells in seawater as measured by quantitative PCR with propidium monoazide. Water Res.

[b6] Bakir MA, Sakamoto M, Kitahara M, Matsumoto M, Benno Y (2006). *Bacteroides dorei* sp. nov., isolated from human faeces. Int J Syst Evol Microbiol.

[b7] Balleste E, Blanch AR (2010). Persistence of Bacteroides species populations in a river as measured by molecular and culture techniques. Appl Environ Microbiol.

[b8] Bernhard AE, Field KG (2000a). A PCR assay to discriminate human and ruminant feces on the basis of host differences in Bacteroides-Prevotella genes encoding 16S rRNA. Appl Environ Microbiol.

[b9] Bernhard AE, Field KG (2000b). Identification of nonpoint sources of fecal pollution in coastal waters by using host-specific 16S ribosomal DNA genetic markers from fecal anaerobes. Appl Environ Microbiol.

[b10] Boehm AB, Fuhrman JA, Mrse RD, Grant SB (2003). Tiered approach for identification of a human fecal pollution source at a recreational beach: case study at Avalon Bay, Catalina Island, California. Environ Sci Technol.

[b11] Boehm AB, Ashbolt NJ, Colford JM, Dunbar LE, Fleming LE, Gold MA, Hansel JA, Hunter PR (2009). A sea change ahead for recreational water quality criteria. J Water Health.

[b12] Bossis R (2011). Application of the SWAT Model to Bacterial Loading rates in Kranji Catchment.

[b13] Bower PA, Scopel CO, Jensen ET, Depas MM, McLellan SL (2005). Detection of genetic markers of fecal indicator bacteria in Lake Michigan and determination of their relationship to *Escherichia coli* densities using standard microbiological methods. Appl Environ Microbiol.

[b14] Cerdeno-Tarraga AM, Patrick S, Crossman LC, Blakely G, Abratt V, Lennard N, Poxton I, Duerden B (2005). Extensive DNA inversions in the B. fragilis genome control. Science.

[b15] Chassard C, Goumy V, Leclerc M, Del'homme C, Bernalier-Donadille A (2007). Characterization of the xylan-degrading microbial community from human faeces. FEMS Microbiol Ecol.

[b16] Chua LHC, Shanahan P, Lo EYM, Shuy EB, Thompson J, Dixon CC, Kerigan KB, Nshimyimana JP (2010). 17th Congress of the Asia and Pacific Division of the International Association of Hydraulic Engineering and Research.

[b17] Converse RR, Piehler MF, Noble RT (2011). Contrasts in concentrations and loads of conventional and alternative indicators of fecal contamination in coastal stormwater. Water Res.

[b18] Dick LK, Stelzer EA, Fong DL, Stoeckel DM (2010). Relative decay of Bacteroidales microbial source tracking markers and cultivated *Escherichia coli* in freshwater microcosms. Appl Environ Microbiol.

[b19] Field R, O'Shea ML (1992). The handling and disposal of residuals from the treatment of urban storm water runoff from separate storm drainage systems. Waste Manage Res.

[b20] Fogarty LR, Voytek MA (2005). Comparison of Bacteriodes-Prevotella 16S rRNA genetic makers for fecal samples from different animal species. Appl Environ Microbiol.

[b21] Gawler AH, Beecher JE, Brandao J, Carroll NM, Falcao L (2007). Validation of host-specific bacteroidales 16S rRNA Genes as markers to determine the origin of fecal pollution in Atlantic Rim countries of the European Union. Water Res.

[b22] Hardina CM, Fujioka RS (1991). Soil: the environmental source of *E. coli* and enterococci in Hawaii's streams. Environ Toxicol Water Qual.

[b23] Harris DC (1995). Quantitative Chemical Analysis.

[b24] Hazen TC (1988). Fecal coliforms as indicators in tropical waters – a review. Tox Assess.

[b25] Horman A, Rimhanen-Finne R, Maunula L, Bonsdorff CHv, Torvela N, Heikinheimo A, Hanninen ML (2004). *Campylobacter* spp., *Giardia* spp., *Cryptosporidium* spp., Noroviruses, and indicator organisms in surface water in southwestern Finland, 2000–2001. Appl Environ Microbiol.

[b26] Jenkins MW, Tiwari S, Lorente M, Gichaba CM, Wuertz S (2009). Identifying human and livestock sources of fecal contamination in Kenya with host-specific Bacteroidales assays. Water Res.

[b27] Johnson JYM, Thomas JE, Graham TA, Townshend I, Byrne J, Selinger LB, Gannon VP (2003). Prevalence of *Escherichia coli* O157: H7 and *Salmonella* spp. in surface waters of southern Alberta and its relation to manure sources. Can J Microbiol.

[b28] Layton BA, Walters SP, Lam LH, Boehm AB (2010). Enterococcus species distribution among human and animal hosts using multiplex PCR. J Appl Microbiol.

[b29] McLain JE, Ryu H, Kabiri-Badr L, Rock CM, Abbaszadegan M (2009). Lack of specificity for PCR assays targeting human Bacteroides 16S rRNA gene: cross-amplification with fish feces. FEMS Microbiol Lett.

[b30] Menon P, Billen G, Servais P (2003). Mortality rates of autochthonous and fecal bacteria in natural aquatic ecosystems. Water Res.

[b31] Miyamoto Y, Watanabe K, Tanaka R, Itoh K (2000). Rapid Identification of human intestinal Bacteroides by 16s rdna-targeted species-specific primers. FEMS Microbiol Lett.

[b32] Noble RT, Fuhrman JA (2001). Enteroviruses detected by reverse transcriptase polymerase chain reaction from the coastal waters of Santa Monica Bay, California: low correlation to bacterial indicator levels. Hydrobiologia.

[b33] Noble RT, Griffith JF, Blackwood AD, Fuhrman JA, Gregory JB, Hernandez X, Liang X, Bera AA (2006). Multitiered approach using quantitative PCR to track sources of fecal pollution affecting Santa Monica Bay, California. Appl Environ Microbiol.

[b34] NTU (2008). Water quality monitoring, modeling and management for Kranji Catchment/Reservoir system – Phases 1 and 2, May 2004 to December 2007.

[b35] Paster BJ, Dewhirst FE, Olsen I, Fraser GJ (1994). Phylogeny of Bacteroides, Prevotella, and *Porphyromonas* spp. and related bacteria. J Bacteriol.

[b36] Pickering AJ, Davis J, Walters SP, Horak HM, Keymer DP, Mushi D, Strickfaden R, Chynoweth JS (2010). Hands, water, and health: fecal contamination in Tanzanian communities with improved, non-networked water supplies. Environ Sci Technol.

[b37] Pruss A (1998). Review of epidemiological studies on health effects from exposure to recreational water. Int J Epidemiol.

[b38] PUB (2011). Active, Beautiful, Clean Waters Design Guidelines.

[b39] Rahman I, Shahamat M, Chowdhury MA, Colwell RR (1996). Potential virulence of viable but nonculturable Shigella dysenteriae type 1. Appl Environ Microbiol.

[b40] Rajal VB, McSwain BS, Thompson DE, Leutenegger CM, Kildare BJ, Wuertz S (2007). Validation of hollow fiber ultrafiltration and real-time PCR using bacteriophage PP7 as surrogate for the quantification of viruses from water samples. Water Res.

[b41] Rivera SC, Hazen TC, Toranzos GA (1988). Isolation of fecal coliforms from pristine sites in a tropical rain-forest. Appl Environ Microbiol.

[b42] Ruimy R, Podglajen I, Breuil J, Christen R, Collatz E (1996). A recent fixation of cfiA genes in a monophyletic cluster of Bacteroides fragilis is correlated with the presence of multiple insertion elements. J Bacteriol.

[b43] Santoro AE, Boehm AB (2007). Frequent occurrence of the human-specific Bacteroides fecal marker at an open coast marine beach: relationship to waves, tides and traditional indicators. Environ Microbiol.

[b44] Sauer EP, VandeWalle JL, Bootsma MJ, McLellan SL (2011). Detection of the human specific Bacteroides genetic marker provides evidence of widespread sewage contamination of stormwater in the urban environment. Water Res.

[b45] Seurinck S, Defoirdt T, Verstraete W, Siciliano SD (2005). Detection and quantification of the human-specific HF183 Bacteroides 16S rRNA genetic marker with real-time PCR for assessment of human faecal pollution in freshwater. Environ Microbiol.

[b46] Shanks OC, Nietch C, Simonich M, Younger M, Reynolds D, Field KG (2006). Basin-wide analysis of the dynamics of fecal contamination and fecal source identification in Tillamook Bay, Oregon. Appl Environ Microbiol.

[b47] Srinivasan S, Aslan A, Xagoraraki I, Alocilja E, Rose J (2011). Escherichia coli, enterococci, and Bacteroides thetaiotaomicron qPCR signals through wastewater and septage treatment. Water Res.

[b48] Surbeck CQ, Jiang SC, Grant SB (2010). Ecological control of fecal indicator bacteria in an urban stream. Environ Sci Technol.

[b49] Thompson JD, Gibson TJ, Plewniak F, Jeanmougin F, Higguns DG (1997). The CLUSTAL_X windows interface: flexible strategies for multiple sequence alignment aided by quality analysis tools. Nucleic Acids Res.

[b50] USEPA (1986). Quality Criteria for Water.

[b51] USEPA (2002a). Implementation Guidance for Ambient Water Quality Criteria for Bacteria.

[b52] USEPA (2009). National Water Quality Inventory: Report to Congress. Vol. EPA 841-R-08–001.

[b53] USEPA (2012). Recreational Water Quality Criteria Office of Water.

[b54] USGS (1999). The Quality of our Nation's waters: Nutrients and Pesticides.

[b55] Van De Werfhorst LC, Sercu B, Holden PA (2011). Comparison of the host specificities of two bacteroidales quantitative PCR assays used for tracking human fecal contamination. Appl Environ Microbiol.

[b56] Walters SP, Field KG (2009). Survival and persistence of human and ruminant-specific faecal Bacteroidales in freshwater microcosms. Environ Microbiol.

[b57] Walters SP, Yamahara KM, Boehm AB (2009). Persistence of nucleic acid markers of health-relevant organisms in seawater microcosms: implications for their use in assessing risk in recreational waters. Water Res.

[b58] Yamahara KM, Walters SP, Boehm AB (2009). Growth of enterococci in unaltered, unseeded beach sands subjected to tidal wetting. Appl. Environ Microbiol.

[b59] Yamahara KM, Sassoubre LM, Goodwin KD, Boehm AB (2012). Occurrence and persistence of bacterial pathogens and indicator organisms in beach sand along the California coast. Appl Environ Microbiol.

[b60] Zhang Y (2011). Water Quality Prediction for Recreational use of Kranji Reservoir, Singapore.

