# Supplementary figures and images for: Distribution and abundance of human-specific Bacteroides and relation to traditional indicators in an urban tropical catchment
Source: J Appl Microbiol. 2014 Feb 25;116(5):1369–83. doi: 10.1111/jam.12455 (PMC4271309; doi:10.1111/jam.12455)

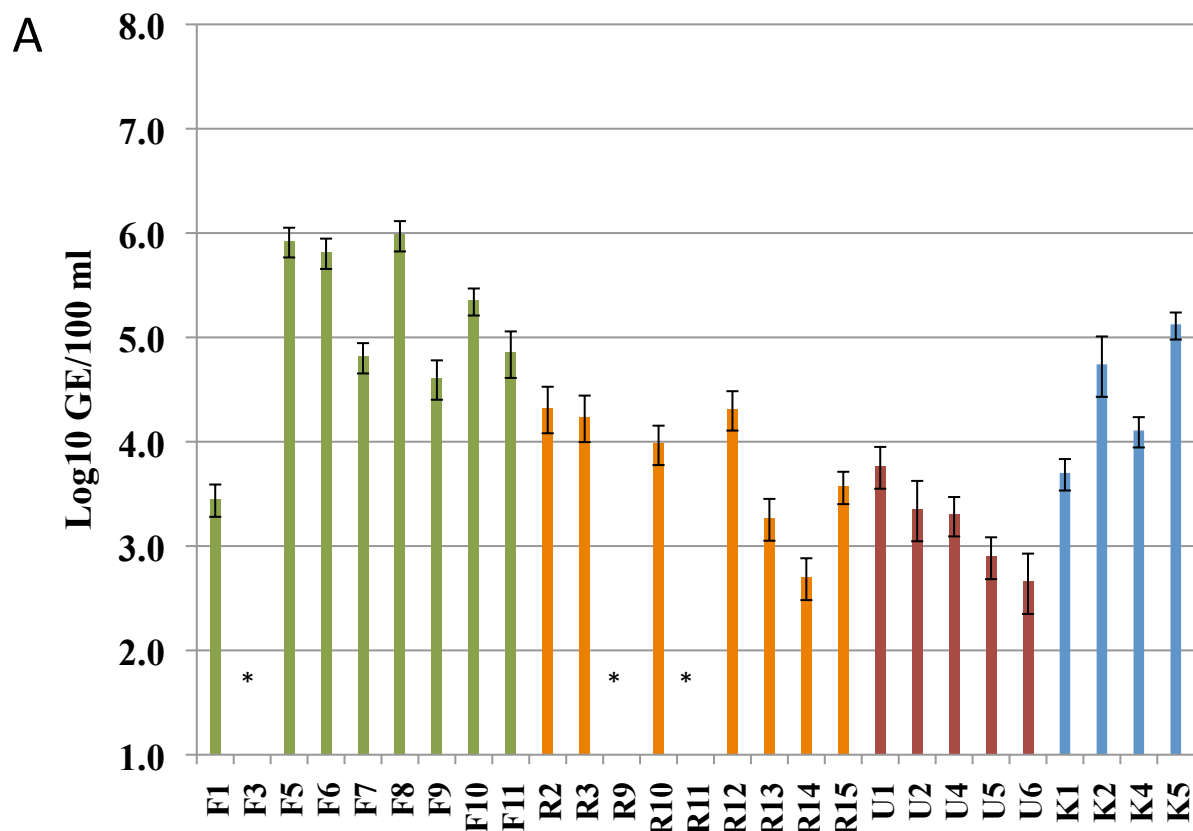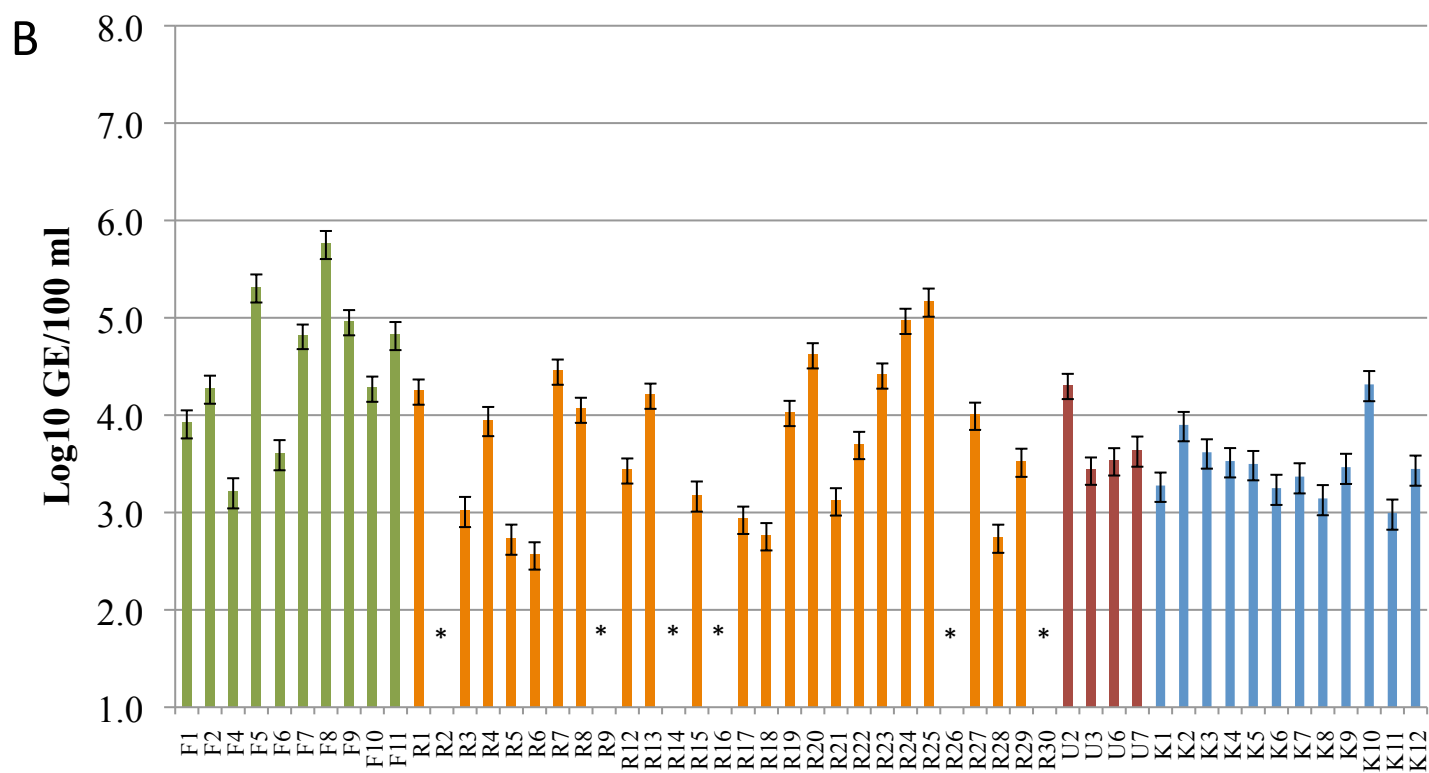

Figure S1

Supplement: Figure S1 — HF183 marker genome equivalents (GE 100 ml−1) in Kranji Reservoir and catchment samples collected in January 2009 (a) and July 2009 (b). *denotes samples with HF levels below detection (i.e. <150 GE 100 ml−1). Sample and colour codes: undeveloped area (U) (purple bars), farming/horticultural area (F) (green bars), residential area (R) (orange bars), Kranji Reservoir (K) (blue bars). Error bars correspond to standard deviations calculated through uncertainty propagation. [file jam0116-1369-SD1.pdf]
