# Supplementary material for: Distribution and abundance of human-specific Bacteroides and relation to traditional indicators in an urban tropical catchment
Source: J Appl Microbiol. 2014 Feb 25;116(5):1369–83. doi: 10.1111/jam.12455 (PMC4271309; doi:10.1111/jam.12455)
Supplement: Supplementary file 3 [file jam0116-1369-SD2.pdf]

Supplementary Table 1. Quantification of Indicator Bacteria in Samples from Kranji Reservoir Catchment, Singapore

**Sample Names**	**Land Use**	**Sampling Dates**	**Dry or wet weather flow**	**HF183 (GE 100ml^{-1})**	**E. coli (MPN 100ml^{-1})**	**Total Coliforms (MPN 100ml^{-1})**
F1	Farming	January 20/2009	Dry	2.84E+03	3.70E+03	1.30E+06
F3	Farming	January 20/2009	Dry	1.50E+02	1.50E+03	1.60E+05
F5	Farming	January 16/2009	Dry	8.43E+05	1.99E+05	1.30E+06
F6	Farming	January 22/2009	Dry	6.58E+05	1.00E+05	7.20E+05
F7	Farming	January 16/2009	Dry	6.56E+04	1.99E+05	1.30E+06
F8	Farming	January 20/2009	Dry	9.69E+05	1.99E+05	1.30E+06
F9	Farming	January 19/2009	Dry	4.08E+04	5.00E+02	9.00E+04
F10	Farming	January 19/2009	Dry	2.27E+05	1.99E+05	1.30E+06
F11	Farming	January 21/2009	Dry	7.15E+04	2.00E+02	8.00E+04
F1	Farming	July 20/2009	Dry	8.36E+03	1.11E+04	5.17E+04
F2	Farming	July 20/2009	Dry	1.90E+04	1.55E+05	1.30E+06
F4	Farming	July 20/2009	Dry	1.64E+03	9.61E+02	2.42E+03
F5	Farming	July 20/2009	Dry	2.08E+05	1.55E+05	1.30E+06
F6	Farming	July 20/2009	Dry	4.04E+03	6.44E+03	1.30E+06
F7	Farming	July 20/2009	Dry	6.61E+04	1.55E+05	1.30E+06
F8	Farming	July 20/2009	Dry	5.82E+05	1.55E+05	1.30E+06
F9	Farming	July 15/2009	Wet	9.24E+04	1.60E+03	2.36E+05
F10	Farming	July 15/2009	Wet	1.92E+04	8.82E+03	1.30E+06
F11	Farming	July 15/2009	Wet	6.75E+04	2.75E+04	3.65E+05
R2	Residential	January 19/2009	Dry	2.11E+04	3.00E+00	1.00E+02
R3	Residential	January 19/2009	Dry	1.74E+04	3.00E+00	1.00E+02
R9	Residential	January 16/2009	Dry	1.50E+02	2.10E+03	1.50E+05
R10	Residential	January 14/2009	Dry	9.64E+03	1.10E+03	1.36E+04
R11	Residential	January 14/2009	Dry	1.50E+02	7.90E+03	1.30E+06
R12	Residential	January 14/2009	Dry	2.06E+04	4.90E+03	5.00E+03
R13	Residential	January 21/2009	Dry	1.87E+03	2.10E+03	1.30E+06
R14	Residential	January 21/2009	Dry	5.04E+02	5.00E+02	3.00E+05
R15	Residential	January 21/2009	Dry	3.76E+03	1.00E+03	1.30E+06
R1	Residential	July 09/2009	Wet	1.79E+04	4.91E+02	5.34E+02
R2	Residential	July 16/2009	Dry	1.50E+02	6.49E+02	2.00E+02
R3	Residential	July 16/2009	Dry	1.05E+03	1.05E+03	1.30E+06
R4	Residential	July 16/2009	Dry	8.95E+03	1.12E+03	4.61E+04
R5	Residential	July 16/2009	Dry	5.47E+02	1.33E+02	3.26E+04
R6	Residential	July 16/2009	Dry	3.72E+02	3.08E+02	1.57E+04
R7	Residential	July/16/2009	Dry	2.87E+04	2.42E+03	2.42E+05
R8	Residential	July 15/2009	Wet	1.16E+04	3.09E+04	3.65E+05
R9	Residential	July 10/2009	Dry	1.50E+02	3.00E+00	1.35E+01
R12	Residential	July 10/2009	Wet	2.77E+03	3.00E+00	1.35E+01
R13	Residential	July 14/2009	Dry	1.62E+04	1.12E+03	1.30E+06
R14	Residential	July 14/2009	Dry	1.50E+02	2.85E+02	2.79E+04
R15	Residential	July 17/2009	Dry	1.52E+03	7.20E+02	3.08E+04
R16	Residential	July 14/2009	Dry	1.50E+02	8.16E+02	1.30E+06
R17	Residential	July 17/2009	Dry	8.65E+02	4.26E+03	2.48E+04
R18	Residential	July 17/2009	Dry	5.86E+02	1.66E+04	7.70E+04
R19	Residential	July 17/2009	Dry	1.08E+04	3.00E+00	8.78E+01
R20	Residential	July 17/2009	Dry	4.22E+04	6.89E+03	5.48E+04
R21	Residential	July 17/2009	Dry	1.34E+03	3.00E+00	1.35E+01
R22	Residential	July 14/2009	Dry	5.07E+03	1.55E+05	1.30E+06
R23	Residential	July 14/2009	Dry	2.62E+04	1.55E+05	1.30E+06
R24	Residential	July 14/2009	Dry	9.54E+04	1.55E+05	1.30E+06
R25	Residential	July 14/2009	Dry	1.49E+05	1.55E+05	1.30E+06

R26	Residential	July 14/2009	Dry	1.50E+02	2.61E+02	1.30E+06
R27	Residential	July 14/2009	Dry	1.01E+04	1.55E+05	1.30E+06
R28	Residential	July 14/2009	Dry	5.59E+02	1.55E+05	2.00E+03
R29	Residential	July 14/2009	Dry	3.37E+03	2.56E+01	1.12E+03
R30	Residential	July 14/2009	Dry	1.50E+02	1.55E+05	1.30E+06
U1	Undeveloped	January 22/2009	Dry	5.88E+03	3.00E+00	1.30E+06
U2	Undeveloped	January 22/2009	Dry	2.27E+03	1.00E+02	8.00E+02
U4	Undeveloped	January 19/2009	Dry	2.00E+03	3.00E+00	3.00E+00
U5	Undeveloped	January 19/2009	Dry	8.00E+02	3.00E+00	5.20E+03
U6	Undeveloped	January 19/2009	Dry	4.57E+02	3.00E+00	2.00E+02
U2	Undeveloped	July 22/2009	Dry	2.05E+04	1.27E+02	1.61E+04
U3	Undeveloped	July 15/2009	Dry	2.77E+03	7.50E+03	4.41E+04
U6	Undeveloped	July 22/2009	Wet	3.45E+03	3.09E+01	3.05E+03
U7	Undeveloped	July 22/2009	Dry	4.39E+03	2.28E+01	3.69E+03
K1	Kranji Reservoir	January 21/2009	Dry	5.02E+03	3.00E+00	3.00E+00
K2	Kranji Reservoir	January 21/2009	Dry	5.51E+04	3.00E+00	3.00E+00
K4	Kranji Reservoir	January 21/2009	Dry	1.28E+04	3.00E+00	3.00E+00
K5	Kranji Reservoir	January 21/2009	Dry	1.33E+05	3.00E+00	3.00E+00
K1	Kranji Reservoir	July 13/2009	Dry	1.89E+03	3.00E+00	1.41E+03
K2	Kranji Reservoir	July 13/2009	Dry	7.94E+03	3.00E+00	7.22E+02
K3	Kranji Reservoir	July 13/2009	Dry	4.16E+03	3.00E+00	1.48E+02
K4	Kranji Reservoir	July 13/2009	Dry	3.38E+03	3.00E+00	1.73E+03
K5	Kranji Reservoir	July 13/2009	Dry	3.15E+03	3.00E+00	1.30E+03
K6	Kranji Reservoir	July 13/2009	Dry	1.78E+03	3.00E+00	5.79E+02
K7	Kranji Reservoir	July 13/2009	Dry	2.34E+03	3.00E+00	2.19E+02
K8	Kranji Reservoir	July 13/2009	Dry	1.39E+03	3.00E+00	4.64E+01
K9	Kranji Reservoir	July 13/2009	Dry	2.92E+03	2.16E+01	6.87E+02
K10	Kranji Reservoir	July 13/2009	Dry	2.07E+04	6.30E+00	1.30E+06
K11	Kranji Reservoir	July 13/2009	Dry	9.91E+02	4.10E+00	1.30E+06
K12	Kranji Reservoir	July 13/2009	Dry	2.80E+03	1.10E+01	1.30E+06
